# Selection of target mutation in rat gastrointestinal tract *E. coli* by minute dosage of enrofloxacin

**DOI:** 10.3389/fmicb.2014.00468

**Published:** 2014-09-04

**Authors:** Dachuan Lin, Kaichao Chen, Ruichao Li, Lizhang Liu, Jiubiao Guo, Wen Yao, Sheng Chen

**Affiliations:** ^1^Food Safety and Technology Research Center, Hong Kong Polytechnic University – Shen Zhen Research InstituteShenzhen, China; ^2^The State Key Lab of Chiroscience, Department of Applied Biology and Chemical Technology, The Hong Kong Polytechnic UniversityKowloon, Hong Kong; ^3^College of Animal Science and Technology, Nanjin Agriculture UniversityNanjin, China

**Keywords:** target mutation, rat GI tract *E. coli*, enrofloxacin, resistance development

## Abstract

It has been suggested that bacterial resistance is selected within a mutation selection window of antibiotics. More recent studies showed that even extremely low concentration of antibiotic could select resistant bacteria *in vitro*. Yet little is known about the exact antibiotic concentration range that can effectively select for resistant organisms in animal gastrointestinal (GI) tract. In this study, the effect of different dosages of enrofloxacin on resistance and mutation development in rat GI tract *E. coli* was investigated by determining the number of resistant *E. coli* recoverable from rat fecal samples. Our data showed that high dose antibiotic treatment could effectively eliminate *E. coli* with single *gyrA* mutation in the early course of treatment, yet the eradication effects diminished upon prolonged treatment. Therapeutic and sub-therapeutic dose (1/10 and 1/100 of therapeutic doses) of enrofloxacin could effectively select for mutation in GI tract *E. coli* at the later course of enrofloxacin treatment and during the cessation periods. Surprisingly, very low dose of enrofloxacin (1/1000 therapeutic dose) could also select for mutation in GI tract *E. coli* at the later course of enrofloxacin treatment, only with slightly lower efficiency. No enrofloxacin-resistant *E. coli* could be selected at all test levels of enrofloxacin during long term treatment and the strength of antibiotic treatment does not alter the overall level of *E. coli* in rat GI tract. This study demonstrated that long term antibiotic treatment seems to be the major trigger for the development of target mutations in GI tract *E. coli*, which provided insight into the rational use of antibiotics in animal husbandry.

## INTRODUCTION

The use of antibiotics in treatment of bacterial infections represents one of the most important inventions in human history. Since its discovery in the 1940s, antibiotics have saved millions of human lives and have also been widely used in the fields of veterinary medicine and agriculture in the past 70 years. However, due to the extensive use and misuse of antibiotics in various settings, most agents have lost their efficacy to bacteria as a result of emergence and spread of multiple-drug-resistant bacterial pathogens (Ferber, 2010). In the last decade, resistance to human first line drugs has increased significantly and the choices of treatment for serious bacterial infections have become extremely limited, threatening to take medicine back into the pre-antibiotic era (Hawkes et al., 2007; Brazier, 2008; Ferber, 2010; Srivastava et al., 2011; Ghafur, 2013).

The mechanisms of antimicrobial resistance in bacteria have been intensively investigated (Tenover, 2006). To date, the mechanisms of bacterial resistance to any antibiotic has been known to a certain extent. However, the driving forces mediating the selection and development of antibiotic resistance in bacteria as well as how resistance genes spread between different organisms and environment niches are complex and still not completely understood. Since resistance to antibiotics in bacteria has been evolving over a long period of time, a complex repertoire of resistance mechanisms have emerged. At this stage, it is still not clear whether mutation development or acquisition of antimicrobial resistance genes is the primary mechanism which is required to initiate the development of antimicrobial resistance in a bacterial population. Nevertheless, a wide range of transferable elements harboring antimicrobial resistance genes is known to be responsible for the rapid dissemination of resistance traits in bacteria (Zhao et al., 2010; Jiang et al., 2011; Zheng et al., 2012; Yang et al., 2014). Regardless of the nature of resistance mechanism, acquisition of antimicrobial resistance genes or genetic mutations responsible for bacterial antibiotic resistance, constant antibiotic pressure is commonly accepted to be the driving force of expansion of the resistant population. It has been suggested that resistant bacteria were selected at the concentration of antibiotics within the mutation selection window, a concentration between the minimal inhibitory concentration (MIC) and minimal mutation prevention concentration (MPC; Zhao and Drlica, 2002; Blondeau, 2009; Firsov et al., 2013). However, recent studies have shown that resistant bacteria can be selected at very low concentration of antibiotics *in vitro*. The concentration can be even lower than the concentration of antibiotics used for growth promotion purpose in animals, and the concentration of environmental antibiotic residues due to natural production by microorganisms and human contamination (Gullberg et al., 2011). However, these studies were conducted in *in vitro* model and could not explain how animal GI tract organisms respond to different concentrations of antibiotics, since growth promotional usage of antibiotics in animals is considered as the most important practice that selected for both antibiotic-resistant genes and bacterial pathogens (Kelly et al., 2004; Marshall and Levy, 2011; Looft et al., 2012). Animal GI tract is considered as a more complicated environment than any other ecosystems. Recent studies have demonstrated the role of bacterial stress responses in antibiotic resistance development (Srinivasan et al., 2012; Bernier et al., 2013; Duval and Lister, 2013). The environmental stresses that the bacteria encounter may lead to variation in physiological functions of the organisms, eliciting changes in the intrinsic mutation rate and abilities to survive drug action (Poole, 2012). Animal GI tract imposes a mixture of stresses to bacteria including low pH, oxidative stress, starvation stress, antimicrobial compounds, and interactions between microbiota, which may influence the development of antibiotic resistance in GI tract flora (Nguyen et al., 2011; Bernier et al., 2013). However, in contrast to *in vitro* condition, the physiological changes and fitness costs arisen during the response to these stresses and the development of mutations may in turn put the GI tract bacteria under adverse conditions which may affect the survival fitness of such organisms in the GI tract environment. The effect of these factors on the development of antimicrobial resistance in GI tract bacteria is not fully understood. In this study, a rat model was used to test the effect of different concentrations of antibiotics on the resistance development in *E. coli* in animal GI tract.

## MATERIALS AND METHODS

### ANTIBIOTIC AND ANIMALS

Enrofloxacin was purchased from Sangon Biotech company (Shanghai, China). The antibiotic was dissolved in 0.1 M NaOH at 10 mg/mL stock solution on the day of use. 11–15 weeks old, specified pathogen-free (SPF) male SD rats with body weight 250–350 g were used in all experiments. Animals were purchased from Guangdong Medical Laboratory Animal Center and were housed individually and allowed free access to food and water. They were examined twice every day for any clinical signs such as behavior, gastrointestinal (GI) function, respiratory distress, food, and water intake etc. The experimental protocol was approved by the Research Animal Care and Use Committee of the Hong Kong Polytechnic University.

### ANTIMICROBIAL TREATMENT

Since fluoroquinolones are concentration-dependent antibiotic (Firsov et al., 2004), we checked the effect of different dosages of fluoroquinolone on the development of resistance to enrofloxacin in *E. coli* in animal GI tracts. Thirty male rats were equally divided into six groups: one group was treated with therapeutic dose of enrofloxacin (10 mg/kg body weight), one was treated with saline as control and other groups were treated with doses of 10-fold, 1/10, 1/100, and 1/1000 of the therapeutic dose.

The oral antibiotic treatment regimen lasted about 1 month including three antibiotic treatments and three cessation gaps between treatments. All the rats were subjected to different doses of enrofloxacin treatment for 5 days, then cessation of antibiotic treatment for 4 days, another enrofloxacin retreatment for 5 days, followed by another cessation of antibiotic treatment for 8 days, then enrofloxacin retreatment for another 5 days, and tracing for 3 more days without antibiotic treatment.

At the indicated time intervals, fresh feces (250–500 mg) were collected and re-suspended in 1 ml of saline. The suspension was mixed and diluted by 10-fold in saline. 100 μl of suspension was plated on MacConkey agar containing 0 mg/L, 0.125 mg/L, 0.5 mg/L, and 2 mg/L of enrofloxacin. The plates were incubated at 37°C for 12 h and the total colony counts were recorded. The colony forming units (CFUs) per gram of feces were determined. Colonies that showed typical morphology of *E. coli* on MacConkey plate (pink to rose-red, large regular colonies) were considered as *E. coli* and some of which were confirmed by 16SrRNA sequencing using primers (F: CCAGACTCCTACGGGAGGCAG, R:CGTATTACCGCGGCTGCTG).

### ANTIMICROBIAL SUSCEPTIBILITY

Antimicrobial susceptibilities to enrofloxacin were determined by the agar dilution method in accordance with Clinical and Laboratory Standards Institute guidelines (CLSI, 2013) using *E. coli* strain ATCC 25922 as quality control. The MICs of enrofloxacin were determined following CLSI guidelines (CLSI, 2013).

### DETERMINATION OF THE TARGET MUTATION IN *E. coli*

Presence of target mutations of the Quinolone-resistance determining regions (QRDR) of the *gyrA* and *parC* genes in *E. coli* were determined by PCR as previously described (Everett et al., 1996).

## RESULTS

Thirty SPF male SD rats were selected for this experiment. Resistance background of GI tract *E. coli* of these rats were checked by plating 200~300 mg of fecal samples of the rats on MacConkey plates with 0, 0.125, 0.5, and 2 mg/L of enrofloxacin. All rats were found to contain a similar amount of *E. coli* in their GI tract (data not shown). Five rats exhibited a background of less susceptible *E. coli* which can grow on MacConkey plate containing 0.5 mg/L of enrofloxacin. No fecal *E. coli* was able to grow on MacConkey plate with 2 mg/L concentration of enrofloxacin (data not shown). Twenty colonies with typical morphology of *E. coli* from MacConkey plates were randomly selected and confirmed to be *E. coli* by 16SrRNA sequencing. Similar *E. coli* confirmation was performed for the following each group of experiment by randomly selecting 20~40 *E. coli* for 16SrRNA sequencing. All the checked colonies were confirmed to be *E. coli* (data not shown).

Upon background check, these rats were separated into six experimental groups. All five rats with a low background of *E. coli* with reduced susceptibility to enrofloxacin were grouped into one category and treated with a high dose of enrofloxacin (10-fold therapeutic dose). The rest of the 25 rats were randomly separated into 5 groups with one control group and 4 different treatment groups including therapeutic dose, 1/10, 1/100, and 1/1000 of the therapeutic dose treatments. The background of the high dose group animals is different from the others. The purpose of the high dose group study is different from other groups and intended to check the efficiency of higher dose of enrofloxacin on the clearance of less susceptible *E. coli* in animal GI tract.

### EFFECT OF HIGH DOSE OF ANTIBIOTIC TREATMENT ON RAT GI TRACT *E. coli*

**FIGURE 1:**
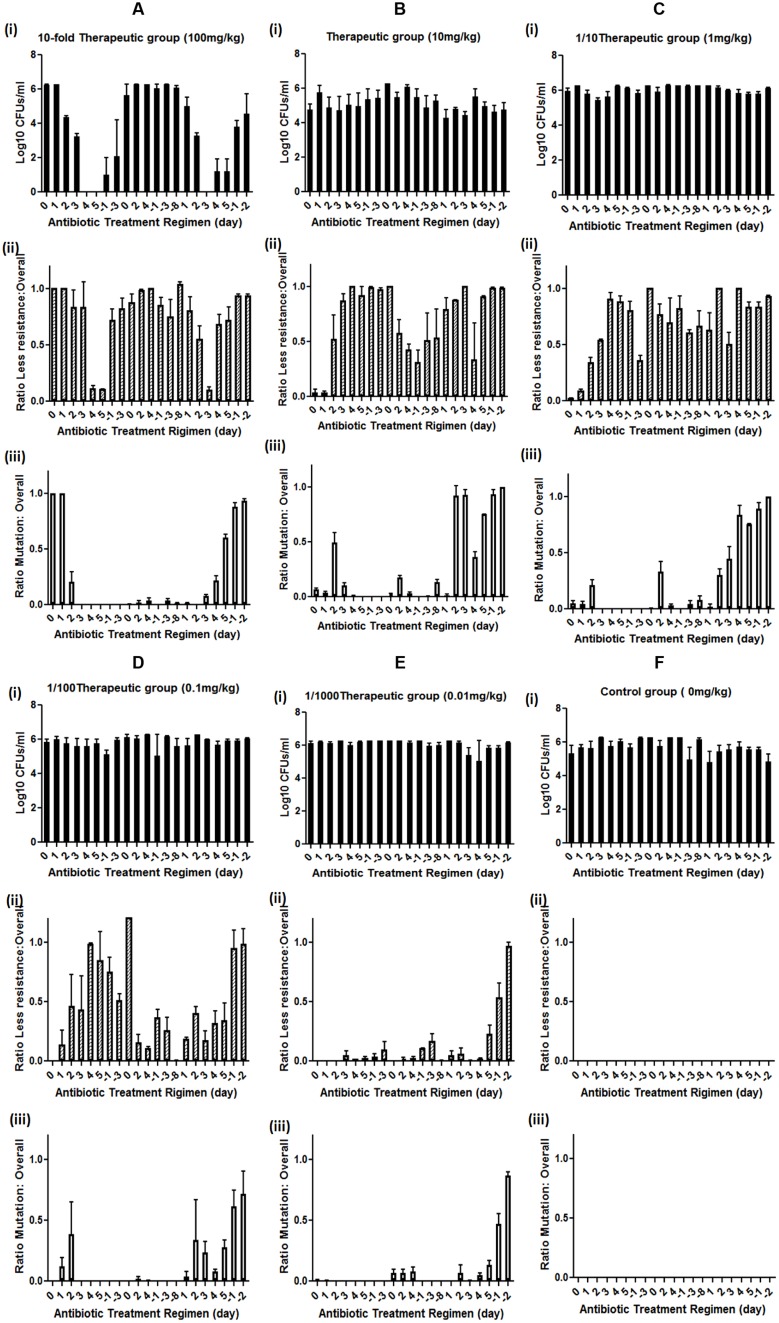
**Effect of different doses of enrofloxacin treatment on mutation development in rat GI tract *E. coli*. (A)** high dose (10-fold therapeutic dose) enrofloxacin treatment, **(B)** therapeutic dose of enrofloxacin treatment, **(C)** sub-therapeutic dose (1/10 of therapeutic dose) of enrofloxacin treatment, **(D)** Low dose (1/100 of therapeutic dose) enrofloxacin treatment, **(E)** very low dose (1/1000 of therapeutic dose) of enrofloxacin treatment, **(F)** Control group; (i) Levels of rat GI tract *E. coli* during enrofloxacin treatment; (ii) levels of less susceptible (grown on MacConkey supplemented with 0.125 mg/L of enrofloxacin) GI tract *E. coli* during enrofloxacin treatment; (iii) Levels of rat GI tract *E. coli* with target mutation (grown on MacConkey supplemented with 0.5 mg/L of enrofloxacin) during enrofloxacin treatment. Y-axis represents the ratio between less susceptible *E. coli* or *E. coli* with target mutation versus overall GI tract *E. coli*. Positive day number represents the antibiotic treatment duration, negative day number represents the duration since the cessation of antibiotic, 0 represents the day of the application of antibiotic and the fecal *E. coli* were isolated before the antibiotic treatment at day 0. The number is the average of data from five rats in the group.

For the 10-fold therapeutic dose treatment group, the application of enrofloxacin caused gradual eradication of rat GI tract *E. coli*. The numbers of GI tract *E. coli* decreased gradually during the first three days of treatment; the reduction became dramatic at the fourth day of treatment and the number of *E coli* recoverable remained at a low level for the first treatment period. After withdrawing the antibiotics, *E. coli* appeared again at the second day after the cessation of antibiotic treatment and increased on the third and fourth day. At the beginning of the second course of treatment period, the numbers of GI tract *E. coli* almost returned back to the normal level. During the whole 5-days antibiotic treatment, the numbers of GI tract *E. coli* were not affected, and remained at the normal level, which lasted throughout the second antibiotic cessation period. The GI tract *E. coli* gradually decreased again upon the start of the third antibiotic treatment. The number of *E. coli* became dramatically reduced again at the third day of the third course of the treatment and then gradually recovered at the fourth and fifth days of the antibiotic treatment. The number of *E. coli* kept recovering upon cessation of antibiotic treatment (**Figure 1Ai**).

The effect of the antibiotic treatment to the development of fluoroquinolone-resistant *E. coli* was recorded. The number of less susceptible *E. coli*, which can grow at MacConkey plate with 0.125 mg/L was high before antibiotic treatment and slightly decreased on the second and third days of antibiotic treatment. The number of less susceptible *E. coli* became dramatically decreased on the fourth and fifth days of treatment. After withdrawing the antibiotic, the number of less susceptible *E. coli* reverted back to the normal level and remained so until the beginning the third course of the antibiotic treatment. The less susceptible *E. coli* slightly decreased at the first day of the third course of the antibiotic treatment and became undetectable at the third day of the treatment, then recovered to normal level throughout the rest of the treatment and non-treatment period (**Figure 1Aii**).

The *E. coli* that can grow on MacConkey with 0.5 mg/L enrofloxacin exhibited different response to high dose enrofloxacin treatment. The numbers of *E. coli* that can grow on MacConkey with 0.5 mg/L enrofloxacin reduced on the seconf day of treatment and became almost undetectable throughout the rest of the first, second and third treatments and non-antibiotic treatment period except for a period of brief appearance during the later course of the third treatment and antibiotic cessation period (**Figure 1Aiii**). Throughout the whole course of treatment, no *E. coli* isolates could be recovered on MacConkey plate with 2 mg/L of enrofloxacin, suggesting that high dose antibiotic could not select for fluoroquinolone-resistant GI tract *E. coli*.

Each of 20 *E. coli* isolates that grew on MacConkey with 0.125 mg/L and 0.5 mg/L enrofloxacin, respectively, were selected to check their MIC and target mutation profiles. *E. coli* that grew on MacConkey with 0.125 mg/L enrofloxacin exhibited a MIC range of enrofloxacin of 0.006~0.03 with no mutation in any of the four target genes *gyrA*, *gyrB*, *parC,* and *parE*; yet all *E. coli* strains that grew on MacConkey with 0.5 mg/L enrofloxacin exhibited MIC of enrofloxacin of 0.25~1 mg/L with single mutation in *gyrA* (S83L; **Table 1**). Therefore, the MacConkey plate with 0.5 mg/L enrofloxacin could be used to check for the mutation rate of GI tract *E. coli* upon antibiotic treatment.

**Table 1:** MIC of enrofloxacin and target mutation profiles of rat GI tract *E. coli*.

Animal group	Origin of *E. coli* (enrofloxacin selective plates)	Number of isolates	MIC range of enrofloxacin (mg/L)	Target mutations	
				*GyrA* (No. of *E. coli*)	*ParC*
A*	0.125 mg/L	20	0.015∼0.06	Wt (20/20)	Wt
	0.5 mg/L	20	0.25∼1	S83L (20/20)	Wt
B	0.125 mg/L	20	0.015∼0.06	Wt (20/20)	Wt
	0.5 mg/L	20	0.25∼1	S83L (20/20)	Wt
C	0.125 mg/L	20	0.015∼0.5	Wt (17/20), S83L (3/20)	Wt
	0.5 mg/L	20	0.25∼1	S83L (20/20)	Wt
D	0.125 mg/L	20	0.06∼0.5	Wt (15/20), S83L (5/20)	Wt
	0.5 mg/L	20	0.125∼1	S83L (20/20)	Wt
E	0.125 mg/L	20	0.06∼1	Wt (11/20), S83L (9/20)	Wt
	0.5 mg/L	20	0.125∼1	S83L (20/20)	Wt

*A, high dose (10-fold therapeutic dose) enrofloxacin treatment; B, therapeutic dose of enrofloxacin treatment; C, sub-therapeutic dose (1/10 of therapeutic dose) of enrofloxacin treatment; D, low dose (1/100 of therapeutic dose) enrofloxacin treatment; E, very low dose (1/1000 of therapeutic dose) of enrofloxacin treatment.

### EFFECT OF THERAPEUTIC DOSE OF ANTIBIOTIC TREATMENT ON RAT GI TRACT *E. coli*

For the therapeutic treatment group, the GI tract *E. coli* exhibited slightly less susceptible *E. coli* background. Hence the therapeutic treatment dose of enrofloxacin did not have any effect on the numbers of *E. coli* in rat GI tract (**Figure 1Bi**). However, after antibiotic treatment, the number of less susceptible *E. coli* decreased slightly and then increased to high level throughout the first course of antibiotic treatment and antibiotic cessation periods. During the second course of treatment, the numbers of less susceptible *E. coli* reduced gradually during treatment and remained at a lower level during the second antibiotic withdrawal period. During the third course of antibiotic treatment, the number of less susceptible *E. coli* increased to a high level and remained so until the end of the experiment (**Figure 1Bii**).

The numbers of *E. coli* that grew on MacConkey agar with 0.5 mg/L enrofloxacin (mutation rate) increased to around half of the total *E. coli* at the second day upon the treatment of enrofloxacin and remained at this level during the first course of the treatment. *E. coli* strains with mutation disappeared during the antibiotic withdrawal period and reappeared during the second course of treatment albeit at a lower level. *E. coli* strains with mutation increased to a high level at the second day of the third course of treatment, then decreased and reappeared again during the antibiotic cessation period (**Figure 1Biii**). The data showed that mutation in *E. coli* might mainly triggered by antibiotic treatment and release of the antibiotic pressure can reduce the numbers of *E. coli* with mutation, which suggested that without antibiotic pressure, the *E. coli* with mutation may be less competitive than other normal *E. coli* in animal GI tract. The long term antibiotic treatment may make *E. coli* with mutation to adapt to animal GI tract, which can be seen from the increased number of *E. coli* with mutation at the end of third course of antibiotic treatment and the following cessation period.

### EFFECT OF SUB-THERAPEUTIC AND LOWER DOSES OF ANTIBIOTIC TREATMENT ON RAT GI TRACT *E. coli*

Similar to the effect of the therapeutic dose of antibiotic treatment, sub-therapeutic dose of enrofloxacin did not have any impact on the overall numbers of *E. coli* in rat GI tract (**Figure 1Ci**). The less susceptible *E. coli* strains emerged upon antibiotic treatment and remained at a stable level throughout the course of the experiment (**Figure 1Cii**). The numbers of GI tract *E. coli* with target mutation slightly increased upon antibiotic treatment and disappeared during the antibiotic cessation period for the first two courses of treatments. The mutation rate was significantly increased in *E. coli* upon the third course of antibiotic treatment (**Figure 1Ciii**).

For the lower-level antibiotic treatment groups, namely 1/100 and 1/1000 of the therapeutic doses, no change in the number of GI tract *E. coli* could be observed throughout the experiment (**Figures 1Di,Ei**). The less susceptible *E. coli* could be selected during the second and third courses of antibiotic treatment and the *E. coli* with mutation could also be selected during second and third courses of the antibiotic treatments, with a higher rate at the end of the third course of the treatment, suggesting long term antibiotic treatment is the major trigger for the development of resistance in GI tract *E. coli* (**Figures 1Dii,Eii**). For the control group, neither less susceptible *E. coli* nor *E. coli* with target mutation could be selected (**Figure 1F**). Throughout the whole experiment, no *E. coli* that can grow on 2 mg/L of enrofloxacin could be obtained (data not shown).

Each of 20 *E. coli* isolates that grew on MacConkey with 0.125 mg/L and 0.5 mg/L enrofloxacin from different treatment experiments were selected to check their MIC and target mutation profiles. Different from the results obtained from the high dose treatment group, *E. coli* that grew on MacConkey with 0.125 mg/L enrofloxacin showed MIC range of enrofloxacin of 0.015~0.5, a little bit higher MIC than *E. coli* from high dose treatment group, with no mutation on any of the four target genes *gyrA*, *gyrB*, *parC,* and *parE* for most of the strains, but not all strains; on the other hand, *E. coli* that grew on MacConkey with 0.5 mg/L enrofloxacin exhibited MIC of enrofloxacin of 0.25~1 mg/L with a single mutation on *GyrA* (S83L) and no mutation at other target genes for all test strains, similar to the results from high dose treatment group (**Table 1**). The results further confirmed the use of MacConkey plate with 0.5 mg/L enrofloxacin as a tool to check for the mutation rate of GI tract *E. coli* upon antibiotic treatment.

## DISCUSSION

Improper uses of antibiotics from clinical applications and promotion of animal growth are the main causes for high prevalence of antimicrobial resistance in bacterial pathogens (Kelly et al., 2004; Marshall and Levy, 2011; Looft et al., 2012). Evidences have shown that in-feed use of antibiotics could dramatically lead to an increase in the number of antimicrobial resistant genes and the size of microbial flora pool in anima GI tract (Looft et al., 2012). Oral usage of antibiotics may be the direct route that facilitates the selection of antimicrobial resistant gene and bacteria pool in animal GI tract (Zhang et al., 2013). These studies reinforced the concept that the antibiotic pressure in animal GI tract favored the amplification of antibiotic-resistant bacterial pool and cause it to become dominant in the animal GI tract, therefore increasing both antibiotic-resistant gene and bacteria pool. This study focuses mainly on the understanding of how GI tract bacteria, in particular *E. coli*, develop resistance upon encountering different levels of antibiotic pressure.

To obtain meaningful interpretation of the data, all rats were checked for the initial load of GI tract *E. coli* and their background level of susceptibility to enrofloxacin. Five rats with a predominant background of low level resistance to enrofloxacin (grow at MacConkey with 0.5 mg/L, but not with 2 mg/L) were arranged into one group and treated with high dose of enrofloxacin to check whether high dose of antibiotic could eradicate organisms with intermediate enrofloxacin resistance, while other groups of mice with lower or no background of less resistant *E. coli* were treated with different doses of antibiotic. This study has come up with several conclusions that may be useful for the understanding of bacterial resistance development in animal GI tract. Firstly, high dose of antibiotic treatment can eradicate less susceptible *E. coli* (with target mutation) and prevent mutation development in *E.coli* in the early course of enrofloxacin treatment, while became less effective for long term treatment; therapeutic dose of enrofloxacin could select for the less susceptible *E. coli* and those containing target mutations. It is commonly accepted that intake of therapeutic dose of antibiotics for the whole course of treatment could prevent the development of resistance in bacterial pathogens. Our data showed that only high dose of antibiotic could effectively eliminate *E. coli* with target mutation, which are present in rat GI tract before treatment. However, therapeutic dose, 1/10 and 1/100 of therapeutic doses of enrofloxacin could select for less susceptible *E. coli* without target mutation and *E. coli* with *de novo* target mutation. Extremely low dose of enrofloxacin treatment, such as 1/1000 of therapeutic dose, can also select for less susceptible *E. coli* without target mutation and *E. coli* with *de novo* target mutation, but with lower efficiency. From the data obtained, we can also see that most of the target mutations in *E. coli* were selected during the later course of antibiotic treatment and antibiotic cessation periods. Long term antibiotic treatment seems to be the major trigger for the development of target mutations in GI tract *E. coli*. Another interesting finding for this study is that antibiotic treatment, except for those with high dosage, did not affect the total number of GI tract *E. coli*. Even under high dosage antibiotic treatment, the number of GI tract *E. coli* decreased upon treatment and then became normal even during the second treatment.

Recent studies have reported the selection of resistant bacteria under very low concentration of antibiotics *in vitro* (Gullberg et al., 2011). However, these studies mainly focus on determining the concentration of antibiotics that allow the resistant bacteria to compete with their drug-susceptible counterparts favorably. In another word, how concentration of antibiotics lower than the MIC of susceptible cells, namely sub-MIC levels, promotes the enrichment of resistant bacteria. One previous study also showed how low concentration of antibiotic selects for *de novo* resistant mutants (Gullberg et al., 2011). The selection process in such study was antibiotic dependent. At 1 μg/ml concentration of streptomycin, *Salmonella* could develop resistance to up to 128 μg/ml of streptomycin after 700 generations, whereas at 2.3 ng/ml of ciprofloxacin, *E. coli* could be selected at 184 ng/ml, but not resistant to ciprofloxacin after 600 generations (Gullberg et al., 2011). Similar to *in vitro* studies, our data showed that regardless of the dosages of the enrofloxacin within three courses of treatment and cessation regimen, no enrofloxacin-resistant (MIC ≥4 mg/L) *E. coli* could be selected. In addition, no double target mutations in *gyrA* or *parC* could be detected in *E. coli* that could grow on MacConkey with 0.5 mg/L enrofloxacin. Our data is consistent with an early study in chicken in which treatment with enrofloxacin at doses routinely prescribed (50 ppm) rapidly reduced the fecal counts of colonized *E. coli* below the detection limit and did not induce resistance, whereas high frequencies of fluoroquinolone-resistant colonized *C. jejuni* were selected due to the *de novo* mutation at the target genes (van Boven et al., 2003). *E. coli* with S83, D87, or both mutations have commonly been reported in *in vitro* selection experiments and contributed to fluoroquinolone resistance in *E. coli* (Vashist et al., 2009). However, in the GI tract, no *E. coli* with resistant to enrofloxacin could be selected and only *E. coli* with reduced susceptibility to enrofloxacin and S^83^F target mutation could be selected, which is probably due to the unique GI environment. These data suggested that the generation of double target mutations in *GyrA* and/or *ParC*, which is the major mechanism of fluoroquinolone resistance in *E. coli*, may be associated with a fitness cost that hampers its survival in animal GI tract (Komp Lindgren et al., 2005; Gualco et al., 2007). In our current research design, we were not able to select enrofloxacin-resistant *E. coli* in mice GI tract. The fact that the high prevalence of fluoroquinolone-resistant *E. coli* in animal GI tract may be due to the long term selection under antibiotic selective pressure in animal gut or colonization of fluoroquinolone-resistant *E. coli* that has been selected outside the animal gut. Lastly, the development of enrofloxacin-resistant *E. coli* in animal GI tract may possibly require the acquisition of plasmid mediated resistance determinant, which may further facilitate the development of target mutation and therefore development of enrofloxacin resistance in *E. coli*. Recent study has shown the high carriage of different PMQR genes in animal *E. coli* isolates, which contribute to the development of enrofloxacin resistance in *E. coli* (Zhao et al., 2010; Yang et al., 2014). Different hypotheses of how GI tact *E. coli* development resistance to fluoroquinolone require further investigations.

In conclusion, this study demonstrated that long term antibiotic treatment seems to be the major trigger for the development of target mutations in GI tract *E. coli*, which provided insights into the rational use of antibiotics in animal husbandry.

## Conflict of Interest Statement

The authors declare that the research was conducted in the absence of any commercial or financial relationships that could be construed as a potential conflict of interest.
