# Supplementary material for: Selection of target mutation in rat gastrointestinal tract E. coli by minute dosage of enrofloxacin
Source: Front Microbiol. 2014 Sep 4;5:468. doi: 10.3389/fmicb.2014.00468 (PMC4154546; doi:10.3389/fmicb.2014.00468)
Supplement: Supplementary file 1 [file Data_Sheet_1.DOC]

**Selection of target mutation in rat gastrointestinal tract E. coli by minute dosage of enrofloxacin**

Dachuan Lin^1^, Kaichao Chen^1^, Ruichao Li^1^, Lizhang Liu^1^, Jiubiao Guo^1^, Wen Yao^2^, Sheng Chen^1^*


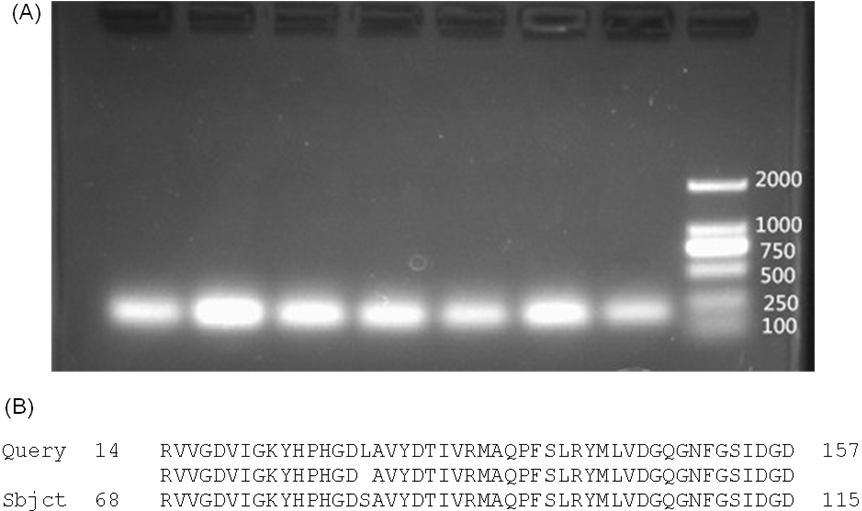


**Supplementary Figure 1.** **PCR and sequencing results for *gyrA* gene from some of the rat GI tract *E. coli* isolates.** (A) gel picture for PCR results of *gyrA* gene from rat GI tract *E. coli* isolates; (B) BLAST of *gyrA* gene sequence from rat GI tract *E. coli* isolate to that of *E. coli* K12 from GenBank. Query is DNA sequence of *gyrA* from rat GI tract *E. coli* isolate, while sbjct is protein sequence of GryA from *E. coli* K12. Mutation S^87^L can be detected from GryA of animal GI tract *E. coli* isolate.
